# Interferons and Dry Eye in Sjögren’s Syndrome

**DOI:** 10.3390/ijms19113548

**Published:** 2018-11-10

**Authors:** Yoko Ogawa, Eisuke Shimizu, Kazuo Tsubota

**Affiliations:** Department of Ophthalmology, Keio University School of Medicine, 35 Shinanomachi, Shinjuku, Tokyo 160-8582, Japan; ophthalmolog1st.acek39@gmail.com

**Keywords:** Sjögren’s syndrome, dry eye, animal model, Interferon-γ

## Abstract

Various cytokines, including interferon (IFN)-γ and IL-17, are augmented, and autoreactive T cells and B cells are activated in the immune pathogenesis of Sjögren’s syndrome (SS). In particular, IFNs are involved in both the early stages of innate immunity by high level of type I IFN in glandular tissue and sera and the later stages of disease progression by type I and type II IFN producing T cells and B cells through B cell activating factor in SS. Genetically modified mouse models for some of these molecules have been reported and will be discussed in this review. New findings from human SS and animal models of SS have elucidated some of the mechanisms underlying SS-related dry eye. We will discuss IFN-γ and several other molecules that represent candidate targets for treating inflammation in SS-related dry eye.

## 1. Introduction

Sjögren’s syndrome (SS) is an intractable autoimmune disease, characterized by chronic lymphocytic infiltration of the lacrimal gland, salivary gland, and other exocrine glands, which lead to dry eye, dry mouth, and extraglandular syndrome [1,2]. 

SS is categorized into primary SS, which is not associated with other autoimmune diseases, and secondary SS, which is associated with other diseases including rheumatoid arthritis, systemic lupus erythematosus, systemic sclerosis, autoimmune liver cirrhosis, and mixed connective tissue disease [3,4]. Moreover, primary SS is further subdivided into the glandular form, which exclusively affects the exocrine glands, and the extraglandular form, which affects organs beyond the exocrine glands [1].

Animal models that reflect clinical findings have been studied to elucidate the pathogenic processes of SS-related dry eye disease, using modern, cutting-edge technology. The comparison of SS animal models to controls has enabled researchers to investigate the detailed mechanisms underlying SS-related dry eye; these pathogenic processes cannot be examined using human samples, which are limited in terms of their availability and applications. On the other hand, the pathophysiology of SS-related dry eye has been controversial because the available animal models do not completely reproduce all of the clinical aspects of dry eye related to SS or its clinical settings [5].

**Figure 1:**
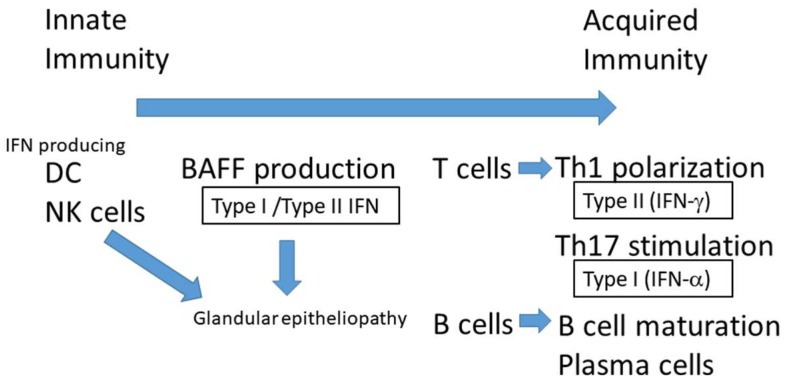
IFNs are involved in both the early stage of innate immunity, in which the type I IFN is elevated in glandular tissue and sera, and the later phase of disease progression, in which type I and type II IFN produce T cells and B cells, stimulated by B-cell-activating factor (BAFF) in SS. Natural killer (NK) cells can activate immature dendritic cells (DCs) through the secretion of IFN-γ. Increasing levels of type II IFNs, such as IFN-γ, are observed in primary SS [2]. The polarization of IFN-γ-secreting Th1 cells is implicated in SS pathogenesis. DC; dendritic cells, NK cells: natural killer cells, IFN; interferon, BAFF: B-cell-activating factor.

Nevertheless, recent advances in basic research have increased our understanding of the dry eye disease caused by SS [5]. It is reported that various cytokines produced by immunocompetent cells including IFN-γ and interleukin (IL)-17 are augmented and autoreactive T cells and B are activated by IFN in the immune pathogenesis of SS exocrine glands [6]. In particular, IFNs are involved in both the early stage of innate immunity, during which the type I IFN is elevated in glandular tissue and sera [7] and the later phase of disease progression, by type I and type II IFN producing T cells and B cells [8], which is stimulated by B-cell-activating factor (BAFF) in SS [2] (Figure 1). BAFF is a cytokine produced by monocytes and dendritic cells (DCs) [9,10] that is crucial for the proliferation, differentiation and survival of B cells [9,10,11]. Genetically modified mouse models for some of these molecules have been reported and will be discussed in this review. New findings from both animal models and human SS have elucidated the mechanisms underlying SS-related dry eye and led to new therapeutic interventions for this disease. 

This review describes recent research on the pathogenesis of SS, focusing on animal models and SS patients. We will highlight IFN-γ and several other molecules as candidates for the treatment of inflammation in SS-related dry eye.

## 2. Interferons

### 2.1. Interferons

IFN-γ, a canonical cytokine, has indispensable roles in innate and adaptive immunity. IFN-γ is primarily secreted from natural killer cells, T cells, and B cells and acts to enhance inflammation [8]. In addition, IFN-γ stimulates macrophages and dendritic cells to attack bacteria and to increase the expression of MHC molecules. IFN-γ is reported to be important for the immune response in autoinflammatory and autoimmune diseases, including SS [8]. 

IFN is produced in response to invasion by viruses, pathogens, and neoplastic cells. While the profile of IFN responsive genes influences the biological pathways leading to IFN production, IFNs also confer a cellular response to invading pathogens, especially against double stranded RNA viruses [7].

IFNs are classified into four types, as follows: (1) type I IFNs, consisting of IFN -α, -β, -ω, -ε, -κ, and -τ and Limitin; (2) type II IFNs, consisting of IFN-γ; (3) the IFN-λ subfamily (type III IFNs), consisting of IFN-λ1, IFN-λ2, IFN-λ3, IL-22, IL-24, and IL-26; and (4) the IL-10 family, consisting of IL-10, IL-19, and IL-20. IFN-γ, the type II IFN, elicits its response through specific receptors, such as IFN-γ receptor 1 (IFNGR1) and IFN-γ receptor 2 (IFNGR2) [12]. When type I IFN binds to the type I IFN receptor (IFN1R), IFNGR1 colocalizes with IFNGR2 and together they induce signal transduction and phosphorylation by IFNGR2, in association with Janus kinase 1/2 (JAK1/2) [13]. IFNs are involved in activating the antiviral response and control immunoreactivity through interactions with their corresponding receptors [14]. It has been reported that a role for IL-12 with IFN-γ, is implicated in primary SS. IL-12 is known to be a potent inducer of Th1 cell polarization. Therefore, IL-12 plays a primary role in increasing the levels of type II IFNs, such as IFN-γ, that are observed in primary SS [2]. 

### 2.2. Involvement of Type I IFN and Type II IFN

Signal transducer and activator of transcription 4 (STAT4) is triggered by a wide range of cytokines, including type I IFN, IL-12 and IL-23, which stimulate Th17 cells and contribute to the regulation of proinflammatory cytokine production. After coupling with receptors, STAT4 is phosphorylated and transferred to the nucleus, leading to the gene transcription of IFN-γ and the activation of Th1 cells. Interestingly, risk alleles in interferon regulatory factor 5 (IRF5) and STAT4 have been shown to be additive in the development of primary SS [15]. 

Discrete expression patterns of the type I and II IFN signatures might be related to distinct SS clinical phenotypes [16]. A recent study revealed that both type I and type II IFN were overexpressed in SS patients versus sicca controls, with the type I IFN signature predominantly observed in peripheral blood and a type II IFN signature predominantly observed in the minor salivary gland (MSG) tissues. In SS-lymphoma minor salivary gland (MSG) tissues, lower levels of IFN-α, but higher levels of IFN-γ and type II IFN-inducible gene (IFIG) transcripts were observed compared to both SS and sicca controls. In a receiver operating characteristic curve analysis, the IFN-γ/IFN-α mRNA ratio in MSG tissues demonstrated the best level of discrimination for lymphoma development [17]. 

BAFF production is highly dependent on type I and type II IFNs. Salivary gland epithelial cells express and secrete BAFF after IFN stimulation, including IFN-α and IFN-γ, suggesting that exocrine gland epithelial cells are important in the pathogenesis of primary SS after stimulation by the innate immunity system [5,16].

Further studies may reveal more details, and the deletion of these regulatory molecules from the endogenous locus may provide clues regarding their functions under physiological conditions. Recent advances in genome editing, such as the clustered regularly interspaced short palindromic repeat CRISPR-Associated Proteins 9 (CRISPR Cas 9) method [18,19], may serve as powerful tools for elucidating these mechanisms in SS-related dry eye animal models and cultured cells from mice and humans.

### 2.3. IFN-γ in SS-Related Dry Eye in Animal Models

IFN-γ is reported to be important in the SS-affected lacrimal gland and ocular surface in several animal models [5]. The expression of autoantigens, including Ro60/Sjögren Syndrome type A antigen (SSA), La/Sjögren Syndrome type B antigen (SSB), α-fodrin, β-fodrin, and the M3 acetylcholine receptor, is thought to be an important trigger for the inflammatory epitheliopathy in SS by mechanism involving clustered differentiation (CD4)^+^ T cell activation [20,21].

The activation of an innate response by IFN producing natural killer cells not only damages the lacrimal gland and the ocular surface but also facilitates antigen presenting cell maturation via IFN-γ [22]. IFN-γ facilitates the shift of naïve CD4^+^ T cells to a T helper type 1 polarization. Pathways and molecules that are genetically associated with SS, including IRF-5 and the IFN signaling pathway, the nuclear factor kappa-light-chain-enhancer of activated B cells (NF-κB), interferon regulatory factor 5 (IRF 5) pathway, the lymphocyte signaling pathway, and antigen presentation machinery [23].

CD25KO model: As abovementioned, IFN-γ, a proinflammatory cytokine, is reported to be critical for lacrimal gland destruction and secretory dysfunction in the CD25KO model of SS [24]. CD25 is the primary receptor for IL-2 is indispensable for the development of regulatory T cells. The CD25KO model mimics an autoimmune disease that is characterized by the spontaneous infiltration of lymphocytes and dacryoadenitis [25].

CD25KO/IL-17AKO double KO mice: An altered balance between IFN-γ and IL-13 in CD25KO/IL-17AKO double KO mice accelerates lacrimal gland destruction by increasing glandular apoptosis and facilitating apoptosis through the increased expression of the IFN-γR in the glandular epithelium and the activation of caspases [24]. These findings suggested that targeting both IFN-γ and IL-17 may be beneficial for treating lacrimal gland inflammation in SS. Related to dry eye evaluation, epidermal growth factor level in tears using enzyme immunosorbent assay is decreased and inflammatory cell infiltration into lacrimal gland were evaluated by histology in this animal model.

CD25/IFN-γ double KO (gammaDKO) mice: A decrease in lacrimal gland destruction in IFN-γ depleted mice has been found to correlate with the degree of T-cell infiltration and the presence of M3R autoantibodies [26]. These reports implicate the significance of IFN-γ in innate immunity and the initiation of this disease. Concerning evaluation for dry eye, lacrimal gland histopathology, immunohistochemistory, gene expression of inflammatory marker, and EGF concentration in tears were analyzed. 

IL-2 knockout (KO), IL-2Rα KO and scurfy mice: Those models are reported to develop the phenotype similar to an autoimmune disease, characterized by the spontaneous infiltration of lymphocytes into exocrine glands and dacryoadenitis, but not scurfy mice. However, adoptive transfer of T cells from Scurfy mice exhibit SS like dacrioadenitits, suggesting that environmental factor may confer the phenotype of SS in Scurfy mice [25]. Pilocalpine-stimulated salivary secretion instead of lacrimal fluid secretion was reduced in IL-2 KO and IL-2Rα KO mice. Regarding scurfy mouse, adoptive transfer of T cells from this mouse induced SS-like phenotype in RAG2 KO mice. Innate immune response inducer, lipopolysaccaridosis (a ligand for toll-like receptor) confer to trigger SS like phenotype. 

Desiccating stress dry eye model: A lymphocyte function-associated antigen-1 (LFA-1) antagonist, named “lifitegrast,” and an anti-intercellular adhesion molecule-1 antagonist have been shown to suppress IFN-γ family genes and improve the conditions of keratoconjunctiva sicca (KCS), including the corneal epithelial barrier dysfunction and goblet cell density/area of ocular surface but not lacrimal gland in a mouse desiccating stress dry eye model that develops KCS similar to SS [27]. Tear secretion level was not tested in this animal model. However, it is important to evaluate the corneal epithelial barrier and goblet cell density both of which influence the severity of dry eye and stability of tear film layer. This report suggested that the IFN-γ family genes and LFA/intercellular adhesion molecule-1 (ICAM-1) signaling are involved in the signal transduction underlying SS-related dry eye.

Non-obese diabetic (NOD) mouse model: Lacrimal gland of this mouse model exhibit inflammatory cell infiltration in pathology. IFN-γ were elevated in lacrimal gland and tears compared to the controls [28]. In cultured lacrimal gland epithelia and corneal cell epithelia, IFN-γ reduce the component of tear secretion including Rab3 D in lacrimal gland and stimulate the expression of MHC class II-mediated antigen presentation. These results suggest the significance that early elevations in IFN-γ levels play in the specific features of dry eye pathology in SS [28]. 

Subtype-3 muscarinic acetylcholine receptors (M3R) KO mice: Tsuboi et al. have reported that retinoic-acid-receptor-related orphan nuclear receptor gamma (ROR-γ) T antagonists suppress SS-like dacryoadenitis through the suppression of IL-17 and IFN-γ production by M3R-specific T cells, suggesting that Th1 cells interact with Th17 cells and collaborate with each other in the development of this disease [29]. CD4^+^ M3R-reactive T cells produce IFN-γ and IL-17 in response to the N-terminal 1 and 1st extracellular loop peptides of M3R, and recombination-activating genes 1 (Rag1) KO mice that received N1- and/or first peptide-immunized splenocytes developed sialadenitis [30]. These reports demonstrate that IFN-γ that is released from autoreactive CD4^+^ T cells by M3R is a critical trigger of local and systemic SS disease manifestations in both innate and adaptive immunity.

MpJ-lpr/lpr mice: On the other hand, MpJ-lpr/lpr mice exhibit a Sjögren’s like syndrome with lymphocytic infiltration in the salivary glands and lacrimal glands, and develop dry eye; they also develop a lupus/autoimmune lymphoproliferative syndrome-like condition. The lacrimal gland lesions in these mice were suggested to be Th2-mediated based on an elevation in IL-4 [31]. In this animal model, the mRNA expression by competitive polymerase chain reaction and immunohistochemistry for frozen section were used to evaluate the inflammatory change in exocrine glands.

### 2.4. Severe Dry Eye Related to Sjögren’s Syndrome 

In SS-associated dry eye, changes or reductions in the epithelial glycocalyx, a loss of goblet cells, and keratinization of the conjunctival and corneal epithelia occur in conjunction with the expression of cornified envelope precursor proteins [5]. Squamous metaplasia frequently occurs where mucosal membrane is transdifferentiated to an epidermalized surface, such as the ocular surface, and this is observed during the transition to severe dry eye in several syndromes, including SS, ocular cicatricial pemphigoid, Stevens Johnson syndrome and graft-versus-host disease (GVHD). Small proline-rich proteins in mice [32], involucrin in human cell line [33] and mice [32], late envelope proteins and filaggrin are reported to be cornified envelope precursor proteins [34,35]. IL-1β and IFN-γ have a primary role in forming the squamous metaplasia on the ocular surface epithelia in response to chronic inflammation. Both cytokines have been shown to be present at the ocular surface in dry eye. The expression of the relevant genes has been shown to precede the squamous phenotype [32]. IFN-γ has been reported to promote goblet cell loss, epithelial apoptosis, and keratinization of the conjunctival epithelium in a dry eye mouse model [36] and is an important contributor to squamous metaplasia in human dry eye disease [24]. IFN-γ upregulates the expression of cornified envelope precursors in keratinocytes [33], corneal epithelial cells [37], and conjunctival epithelial cells from patients with SS [34,38]. During the process of squamous metaplasia, IFN-γ is released by infiltrating Th1 cells and NK cells at the ocular surface [31].

### 2.5. The Microbiome in SS-Related Dry Eye and IFN-γ Level 

Recent research suggests that the microbiome is related to the immune response in SS-associated dry eye. Previously, dysbiosis was suspected to be involved in the pathogenesis of SS, similar to other autoimmune diseases, such as inflammatory bowel disease, Crohn’s disease, and rheumatoid arthritis [39]. Recent studies demonstrated that an alteration of the microbiome may decrease the IFN-γ levels in SS [40]. Furthermore, Zaheer et al. examined CD25KO mice, which develop SS-like inflammation, in a germ-free environment. They found that germ-free CD25KO mice expressed that higher levels of IFN-γ and IL-12 than did conventional CD25KO mice. In addition, the expression levels of IFN-γ, IL-12, and other inflammation-related molecules improved after the germ-free CD25KO mice received fecal microbiota transplantations from conventional mice [41]. These results demonstrated that the microbiome plays an important role in SS development. However, most of the mechanisms behind these changes are unknown, and further studies are needed to investigate the relevance of the microbiome in the molecular mechanisms of autoimmune disease.

### 2.6. Sjögren’s Syndrome-Related Dry Eye in Humans

Studies on human SS revealed diffuse lymphocyte infiltration of the acinar areas of the lacrimal gland and conjunctiva. B cells were the predominant infiltrating cell type, but other cells, including activated CD4^+^ T cells and CD8^+^ T cells with HLA-DR and costimulatory molecules, were found with glandular epithelial cells and stromal infiltrating cells in SS patients, while T cell dominant characteristics were found in the lacrimal glands from patients with GVHD [42,43]. Clusters of plasma cells were frequently observed in the interlobular areas of the lacrimal gland and the conjunctival stroma of patients with SS, suggesting that abnormal antibody production and tissue damage in the local microenvironment of the ocular surface and the lacrimal gland were caused by SS. However, there are limitations when studying clinical samples, in terms of comparison with controls and examining the mechanisms of the pathogenic process of SS in the lacrimal gland and on the ocular surface. 

Environmental factors including viruses activate epithelial cells of exocrine gland and dendritic cells, which produce proinflammatory cytokines like IL-17 and IFN-γ. Glandular epithelial cells produce chemokines such as chemokine (C–X–C motif) ligand (CXCL) 9 and CXCL10 [44]. 

Recently, it is reported that H1N1 vaccination in SS patient promote polyclonal B cell activation and autoantibody production through type I IFN [45]. Considering hyperreactive B cells in type I IFN milieu, we need to pay much attention to vaccination in SS patients. 

A Sjögren’s syndrome susceptibility locus at OAS1 was identified in responding type I IFN [46] using the 2002 American-European Consensus Group (AECG) Criteria with dry eye symptom and/or ocular surface staining and/or reduced tear production [47] and need to be re-evaluated by the newly proposed 2016 American College of Rheumatology (ACR)/European League Against Rheumatism (EULAR) criteria [4] to compare and confirm this susceptibility locus. Based on the heterogeneity of the clinical presentation and pathology in SS, the IFN expression patterns is likely to vary among individuals [16] and the systemic IFN type I and type II signature influence the disease severity [48]. IFN-γ, a type II IFN, is involved in the most frequent pathways that have been identified in the pathogenic processes of human SS [16], although three distinct patterns of IFN were evident: type I-predominant, type II-predominant, and type I/II mixed IFN among those patients. The authors evaluated MSG, however minor salivary gland severity well reflect the ocular surface disturbance in SS patients. IFN-γ facilitates naïve CD4 T cells to shift into a T helper type 1 polarization. Genetic associations with SS, including interferon regulatory factor-5 (IRF-5) and the IFN signaling pathway, have been suggested [23]. 

### 2.7. Treatment for SS Related Dry Eye Patients

Aqueous-deficient dry eye in SS is treated with tear lubrication using artificial tears, mucin-producing eye drops, punctal plug, or surgical occlusion depending on the severity of the dry eye. For SS patients, it is essential to use preservative-free topical reagents [49]. 

The mucin-producing eye drops diquafosol and rebamipide are reported to be effective for SS dry eye [50,51,52,53,54]. Diquafosol is a dinucleotide derivative that has purinoreceptor P2Y2 receptor agonist activity. It stimulates P2Y2 receptors at the ocular surface, to produce tears and mucin secretion from the conjunctiva through an elevated intracellular calcium concentration [55,56]. Rebamipide, a mucosal protective and anti-inflammatory agent, is also recommended for SS dry eye [38,50,51]. Rebamipide has anti-inflammatory effects through CD4 inhibition and macrophage activation [57]. 

Systemic corticosteroid is not recommended for SS dry eye and dry mouth [1]. Topical cyclosporine 0.05% (Restasis) is approved for treating dry eye by the FDA and the European Medicine Agency (EMA) [58]. Ikervis, cyclosporine cationic emulsion, has been approved by the EMA [59] and in some Asian countries [60]. Recently, topical lifitegrast, an integrin inhibitor, a LFA-1 antagonist, was approved by the FDA for the treatment of dry eye [61]. Recently, protocols for tear film-oriented therapy has been proposed by the Japan Dry Eye Society, which recommended low-dose corticosteroid therapy for the anti-inflammatory treatment of dry eye disease (http://www.dryeye.ne.jp/tfot/index.html). (access on 9 November 2018).

SS dry eye frequently involves evaporative in addition to aqueous-deficient dry eye. Therefore, treatment of the meibomian gland and eye lid is often required. Warm compression, lid hygiene, topical and oral antibiotics, and oil-containing eye drops are helpful to improve this type of dry eye [62]. Omega 3-containing fish oil is effective for SS-related meibomian gland dysfunction (MGD) [63].

Rituximab, a chimeric monoclonal antibody targeting the pan-B-cell marker CD20, and abatacept, a immunomodulatory drug that inhibit T-cell activation via co-stimulatory blockade, are recommended biological reagents for systemic SS in the Japanese SS clinical practice guidelines [1] and may improve SS dry eye [64].

Although an IFN-based therapeutic strategy has not yet been approved, there are accumulating evidence in humans supports it [65]. Considering the mechanistic insight that IFN signaling is involved in SS related dry eye in mice and humans, the development of IFN-related therapeutic interventions could be indispensable treating SS related dry eye.

### 2.8. Hypothetical Pathogenic Processes of SS-Related Dry Eye 

**Figure 2:**
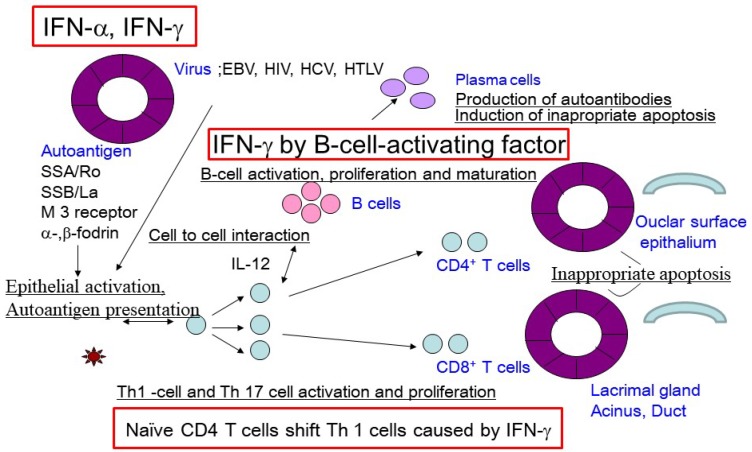
Hypothetical pathogenic process of Sjögren’s syndrome related dry eye diseasemodified form Sumida, T. et al [67].Viral infection acts as a trigger for the autoimmune response in SS. SS-associated ribonucleoprotein autoantigens, including Ro60/SSA, La/SSB, α-fodrin, β-fodrin, and M3 muscarinic acetylcholine receptor, are released by epithelial activation and damage. Epithelial cells are activated by IFN signaling pathways, followed by the subsequent infiltration of lymphocytes, primarily T cells, into the lacrimal glands and the secretion of B-cell-activating factor by epithelial cells. The subsequent B-cell activation and maturation as plasma cells results in the secretion of altered autoantibodies. These processes stimulate a chronic immune cascade, leading to lacrimal gland and ocular surface dysfunction and thus to dry eye. EBV; Epstein‒Barr Virus, HIV; Human Immunodeficiency Virus, HCV; Hepatitis C Virus, HTLV; Human T-lymphotrophic Virus.

Viral infections, such as Epstein‒Barr virus, HIV, hepatitis C virus, coxsackie virus, or human trophic lymphocyte virus type 1 (HTLV-1), of glandular epithelial cells have been reported to trigger the autoimmune response in SS [2,66]. Epithelial activation and damage due to the viral infection could lead to the release of SS-associated ribonucleoprotein autoantigens, including Ro60/SSA, La/SSB, and supporting and organizing scaffold proteins for cell membranes, such as α-fodrin, β-fodrin, and the M3 muscarinic acetylcholine receptor, and Th1-related chemokines, such as chemokine (C–X–C motif) ligand 1 (CXCL1) and CXCL9, in exocrine glands [67]. Epithelial cell activation by viral infection may be promoted in individuals who carry specific alleles of genes that encode proteins in the IFN signaling pathways [2]. The subsequent secretion of proinflammatory cytokines, including IFN-γ, may promote the infiltration of lymphocytes, primarily T cells, into the lacrimal glands, and increase BAFF secretion by epithelial cells [10]. These conditions promote B-cell activation and maturation as plasma cells and the secretion of altered autoantibodies, especially in SS-susceptible individuals. IFN-γ may be associated with the maturation of B cells into plasma cells and the production of altered antibodies. Alternatively, abnormally accumulated autoantibodies can form immune complexes with autoantigens, which participate in the local microenvironment of the salivary and lacrimal glands, as well as in the conjunctival mucosal membrane. Collectively, these processes stimulate the activation of a chronic immune cascade, leading to dry eye caused by lacrimal gland and ocular surface dysfunction (Figure 2). 

## 3. Future Directions

It will be important to carefully evaluate and confirm the appropriateness and accuracy of of the various mouse models for SS and the significance of the data obtained from studies of these mice, to better understand the human disease and potential interventional therapies.

There are many unresolved issues associated with SS-related dry eye, including the pathogenic process, the underlying mechanisms, and possible clinically translatable therapeutic interventions. Examining these issues using appropriate animal models of SS-related dry eye, may lead to the development of specific and precise therapeutic interventions to alleviate this intractable disease.
